# Immunogenicity of an oral rotavirus vaccine administered with prenatal nutritional support in Niger: A cluster randomized clinical trial

**DOI:** 10.1371/journal.pmed.1003720

**Published:** 2021-08-10

**Authors:** Sheila Isanaka, Souna Garba, Brian Plikaytis, Monica Malone McNeal, Ousmane Guindo, Céline Langendorf, Eric Adehossi, Iza Ciglenecki, Rebecca F. Grais

**Affiliations:** 1 Department of Research, Epicentre, Paris, France; 2 Departments of Nutrition and Global Health and Population, Harvard T.H. Chan School of Public Health, Boston, Massachusetts, United States of America; 3 Epicentre, Niamey, Niger; 4 BioStat Consulting, LLC, Worthington, Ohio, United States of America; 5 Department of Pediatrics, University of Cincinnati, Division of Infectious Diseases, Cincinnati Children’s Hospital Medical Center, Cincinnati, Ohio, United States of America; 6 National Hospital, Niamey, Niger; 7 Médecins Sans Frontières—Operational Center Geneva, Geneva, Switzerland; Makerere University Medical School, UGANDA

## Abstract

**Background:**

Nutritional status may play a role in infant immune development. To identify potential boosters of immunogenicity in low-income countries where oral vaccine efficacy is low, we tested the effect of prenatal nutritional supplementation on immune response to 3 doses of a live oral rotavirus vaccine.

**Methods and findings:**

We nested a cluster randomized trial within a double-blind, placebo-controlled randomized efficacy trial to assess the effect of 3 prenatal nutritional supplements (lipid-based nutrient supplement [LNS], multiple micronutrient supplement [MMS], or iron–folic acid [IFA]) on infant immune response (*n* = 53 villages and 1,525 infants with valid serology results: 794 in the vaccine group and 731 in the placebo group). From September 2015 to February 2017, participating women received prenatal nutrient supplement during pregnancy. Eligible infants were then randomized to receive 3 doses of an oral rotavirus vaccine or placebo at 6–8 weeks of age (mean age: 6.3 weeks, 50% female). Infant sera (pre-Dose 1 and 28 days post-Dose 3) were analyzed for anti-rotavirus immunoglobulin A (IgA) using enzyme-linked immunosorbent assay (ELISA). The primary immunogenicity end point, seroconversion defined as ≥3-fold increase in IgA, was compared in vaccinated infants among the 3 supplement groups and between vaccine/placebo groups using mixed model analysis of variance procedures. Seroconversion did not differ by supplementation group (41.1% (94/229) with LNS vs. 39.1% (102/261) with multiple micronutrients (MMN) vs. 38.8% (118/304) with IFA, *p* = 0.91). Overall, 39.6% (*n* = 314/794) of infants who received vaccine seroconverted, compared to 29.0% (*n* = 212/731) of infants who received placebo (relative risk [RR]: 1.36; 95% confidence interval [CI]: 1.18, 1.57, *p* < 0.001). This study was conducted in a high rotavirus transmission setting. Study limitations include the absence of an immune correlate of protection for rotavirus vaccines, with the implications of using serum anti-rotavirus IgA for the assessment of immunogenicity and efficacy in low-income countries unclear.

**Conclusions:**

This study showed no effect of the type of prenatal nutrient supplementation on immune response in this setting. Immune response varied depending on previous exposure to rotavirus, suggesting that alternative delivery modalities and schedules may be considered to improve vaccine performance in high transmission settings.

**Trial registration:**

ClinicalTrials.gov NCT02145000.

## Abstract 2

Sheila Isanaka and co-workers study nutrient supplementation and infants’ immune responses to rotavirus vaccination.

## Author summary

**Why was this study done?:**

- The World Health Organization recommends that all children be vaccinated against rotavirus, the most common cause of severe childhood diarrhea and a major cause of morbidity and mortality worldwide.
- Two oral rotavirus vaccines have been shown to be highly efficacious against childhood diarrhea in high- and middle-income countries (85%–98%), but, virtually, all trials in sub-Saharan Africa and South Asia have shown lower immune response and much lower efficacy (40%–64%), leaving many children unprotected.
- As the development of the immune cells that support vaccine response specifically depend on an adequate supply of micronutrients, nutritional supplementation has been considered as a potential adjunctive intervention to improve oral vaccine performance.

**What did the researchers do and find?:**

- We nested a cluster randomized trial within a double-blind, placebo-controlled randomized efficacy trial to assess the effect of 3 prenatal nutritional supplements (lipid-based nutrient supplement [LNS], multiple micronutrient supplement [MMS], or iron–folic acid [IFA]) on infant immune response.
- This study found no effect of the type of prenatal nutrient supplementation on immune response in this setting; however, the immune response appeared to vary by previous exposure to rotavirus.

**What do these findings mean?:**

- To improve the oral vaccine immunogenicity gap between low- and high-income countries, a more complete understanding of the factors influencing immunogenicity and a reliable correlate of protection are needed.
- Without improvement in oral vaccine performance, alternative delivery modalities and schedules may be considered to improve vaccine performance in high transmission settings.

## Introduction

Oral vaccines offer several advantages over parenteral vaccines: They can be produced in large quantities at low cost, are easy to administer, and can effectively induce local immunity in the intestinal mucosa to block disease transmission [1]. There are currently 4 licensed oral vaccines against rotavirus and others in development. The impact of oral rotavirus vaccines was swiftly shown following introduction in national immunization programs with demonstrated reductions in rotavirus disease, hospital admissions, and mortality [2].

Oral vaccines in general, however, are only half as effective in low-income countries, where child mortality is high, and disease burden is greatest, compared to high-income countries. This gap in oral vaccine efficacy was first observed in the 1950s with the introduction of the oral polio vaccine (OPV) [3] and has been observed for other live oral vaccines such as typhoid and cholera [4,5]. Oral rotavirus vaccines are 85% to 98% efficacious against severe rotavirus gastroenteritis (SRVGE) among North American and European infants [6,7] but only 40% to 64% in sub-Saharan Africa [8–10].

The reasons for lower oral vaccine efficacy in low-income countries are not well understood [11], but early evidence suggests that nutritional status, even in early life, may play a role in immunity [12]. Consequently, nutritional supplementation has been considered as a potential intervention to improve oral vaccine performance [13].

In recognition of the need to identify potential boosters of immunogenicity in low-income countries where oral vaccine efficacy is low, we nested a cluster randomized study within a double-blind, placebo-controlled randomized phase III efficacy trial to test the effect of the type of prenatal nutritional supplementation on immune response to 3 doses of a live oral rotavirus vaccine in Niger.

## Methods

### Study site

The study was conducted in the Madarounfa Health District, Niger from August 2014 to December 2019. Niger is one of the poorest countries in the world, ranking 189 of 189 in 2019 on the Human Development Index. The Madarounfa Health District is rural and representative of the Sahel region. Average fertility is high (7.1 live births per female[14]), as is infant mortality (84 deaths per 1000 live births[15]). Maternal malnutrition in pregnancy is similarly high, with 13% of expecting mothers having a low pre-pregnancy body mass index [14].

### Study design

We conducted a double-blind, placebo-controlled randomized Phase III trial to assess the efficacy of Rotasiil (Serum Institute of India), a live oral rotavirus vaccine, to prevent SRVGE. The efficacy study design and procedures have been published previously [16]. The primary results showed a per protocol efficacy of 66.7% (95% confidence interval [CI]: 49.9, 77.9). A cluster randomized trial was nested within the parent trial to assess the effect of the type of prenatal nutritional supplementation on immune response. The unit of randomization to prenatal supplementation was the village, in which all pregnant women in a participating village received the same nutrient supplement during pregnancy to reduce the likelihood of contamination.

The trial was conducted in accordance with Good Clinical Practice guidelines (ClinicalTrials.gov Identifier: NCT02145000).The study protocol was approved by the ethics committee of the World Health Organization (Geneva, Switzerland), the Western Institutional Review Board (Olympia, Washington, United States of America), Comité Consultatif National d’Ethique (Niamey, Niger), the Comité de Protection des Personnes (Ile-de-France XI, France), and Hôpitaux Universitaires de Génève (Geneva, Switzerland). A Consolidated Standards of Reporting Trials (CONSORT) checklist is available (see S1 Protocol, S1 Statistical Analysis, and S1 CONSORT Checklist).

### Randomization and masking

A total of 53 villages attached to a study health center were randomly assigned to one of 3 prenatal nutrient supplements (lipid-based nutrient supplement [LNS], multiple micronutrient supplement [MMS], or iron–folic acid [IFA]) in a 1:1:1: ratio, stratified by village size. Randomized village assignment was made by the head of each village who selected the name of one of 3 supplements from a jar after providing consent for village participation. Individual inclusion in the immunogenicity sub-study was determined by a 2-stage enrollment process for the mother–infant pair. First, pregnant women were identified and provided written informed consent for the prenatal supplementation and follow-up until 6 months postpartum. Second, at 6 to 8 weeks of age, infants of participating women were evaluated for eligibility in the parent trial, and, if eligible, randomized to vaccine or placebo and follow-up up to 2 years of age as per the parent trial protocol. Successive infants whose mothers completed nutrient supplementation were enrolled in the immunogenicity sub-study until the target sample size for the immunogenicity analysis was achieved. Enrollment was continuous throughout the calendar year, as rotavirus is known to circulate year round in Niger [17].

Village assignment to the prenatal supplement was open as it was not possible to blind participants or study staff to type of supplement received. Individual assignment to vaccine or placebo was blinded to participants and study staff for the whole study period. Vaccine and placebo packaging were labeled with identical presentations and were indistinguishable.

### Procedures

All nonpregnant women of reproductive age in participating villages were asked for written informed consent to participate in monthly pregnancy surveillance. Women with a pregnancy confirmed by urine test (Wondfo Biotech, Guangzhou, China) were eligible for prenatal supplementation if ≤30 weeks gestation using the date of last menstrual period and intending to remain in the study area for delivery and 2 years thereafter. Pregnant women were excluded if having a need for frequent medical attention due to a chronic condition or hospitalization due to severe illness, history of allergy to peanuts, or pregnancy complications evident at enrollment (moderate to severe edema, blood hemoglobin <7 g/dL, or diastolic blood pressure >90 mm Hg). If a woman was found eligible, a study midwife conducted a physical and obstetric exam, and standard diagnostic and therapeutic services for pre- and postnatal care up to 6 months postpartum were provided as per national guidelines. Breastmilk (10 mL) samples were collected at 6 weeks and 6 months postpartum.

Daily nutritional supplements were provided at home on a weekly basis from enrollment until pregnancy outcome. IFA (Remedica, Limassol, Cyprus) represents usual standard of care during pregnancy as per the national guidelines of Niger and was considered the control group in this setting. Multiple micronutrients (MMN) included 22 micronutrients at 2 times the recommended daily allowance (RDA) where possible, based on evidence showing that a daily supplement of twice the RDA was more effective in terms of improving birth outcomes among women in Guinea-Bissau [18] (DSM Nutritional Products, Isando, South Africa). LNS was a 40-g formulation of a ready-to-use food made of peanuts, oil, dried skimmed milk powder, and sugar that was specifically designed for use in pregnancy [19] and provided the same level of micronutrients provided in the MMN arm with the addition of energy, protein, and lipids (Nutriset, Malaunay, France). The composition of the 3 nutrient supplements is provided in S1 Table.

All infants born to women enrolled in the prenatal nutrition intervention were individually evaluated for eligibility in the parent trial at 6 to 8 weeks of age, as per the specific inclusion and exclusion criteria outlined in the parent trial [16]. Eligible infants received 3 doses of vaccine or placebo at 6, 10, and 14 weeks of age. To assess the serum immune response, a subsample of infants provided venous blood (2 mL) samples before Dose 1 and 28 days post-Dose 3. Routine vaccines administered through the Expanded Program on Immunization (EPI) were concomitantly administered with the vaccine or placebo. No specific instructions about breastfeeding were given around the time of administration. Enrolled infants participated in all follow-up and surveillance for gastroenteritis and adverse events as per protocol of the parent trial until 2 years of age.

All blood samples were collected at the health facility and transported on the same day in freezer packs at 2 to 8°C to the Epicentre laboratory in Maradi, where they were stored at −80°C until shipment for analysis. Infant sera and breastmilk samples collected for immunological analysis were isolated and stored at −80°C until shipped for analysis at Cincinnati Children’s Hospital Medical Center Laboratory for Specialized Clinical Studies (Cincinnati, Ohio, USA). Enzyme-linked immunosorbent assay (ELISA) was used as previously described [16] to detect and quantify anti-rotavirus immunoglobulin A (IgA) and immunoglobulin G (IgG) (pre-Dose 1 sample only) concentrations (AU/mL). The lower limit of detection of the assay was 7.5 AU/mL. If rotavirus IgA was not detected in a sample, the concentration assigned corresponded to the lower limit (i.e., 7.5 AU/mL). IgG at pre-Dose 1 was considered to represent maternal IgG or natural infection.

### Statistical analysis

The primary analysis population to evaluate the effect of the type of nutritional supplement on immunogenicity included infants whose mothers completed the prenatal supplementation per protocol received 3 doses of vaccine/placebo per protocol of the parent trial and had valid serology results at Dose 1 and 28 days post-Dose 3. To assess the effect of prenatal nutritional supplementation type on immune response to Rotasiil, 660 children (*n* = 220 per prenatal nutritional group) receiving vaccine were needed to provide 90% power to detect 20% absolute difference in the proportion of children that seroconvert between nutritional interventions, assuming a seroconversion rate of 30% among those receiving IFA, 20% non-accessibility (including withdrawal and loss to follow-up), 30% exclusion due to detection of rotavirus disease between vaccine doses, and a design effect of 1.2 to account for the cluster randomized design in the absence of data on the intracluster correlation. The primary analysis population for immunogenicity included infants who received 3 doses of vaccine/placebo per protocol of the parent trial and had valid serology results at Dose 1 and 28 days post-Dose 3. Assuming a seroconversion rate of 30% in the placebo group, 20% non-accessibility, and 30% exclusion due to detection of rotavirus disease between vaccine doses, the sample of 1,320 infants (*n* = 660 per group) required to evaluate the effect of supplement type among vaccinated children provided >90% power to detect a 20% absolute difference between the vaccine and placebo groups in the proportion of children that seroconvert.

The primary outcome was seroconversion, defined as ≥3-fold increase in IgA from Dose 1 to 28 days post-Dose 3. The proportion of infants seroconverting was calculated with corresponding 95% CIs and compared in vaccinated infants among the 3 supplement groups using a mixed model binomial regression with a random effect for the village. Geometric mean concentrations (GMCs) and CIs were compared among the 3 supplement groups using mixed model analysis of variance procedures with random effects for the individual, village, and health centers. When the supplement group was significant at *p* ≤ 0.05, linear contrasts were performed to compare MMS and LNS to IFA.

The proportion of infants seroconverting and GMC were compared between the vaccine and placebo groups using the methods described above. We considered potential modification of the vaccine versus placebo effect by maternal breastmilk IgA pre-Dose 1, child serum IgG pre-Dose 1, children serum IgA ± 20 AU/ml pre-Dose 1, child sex, concurrent OPV administration, birth weight, maternal supplementation group, and season of administration of Dose 1 using the likelihood ratio test. Data analysis was conducted using SAS software (version 9.4, SAS Institute, North Carolina, USA).

## Results

**Fig 1:**
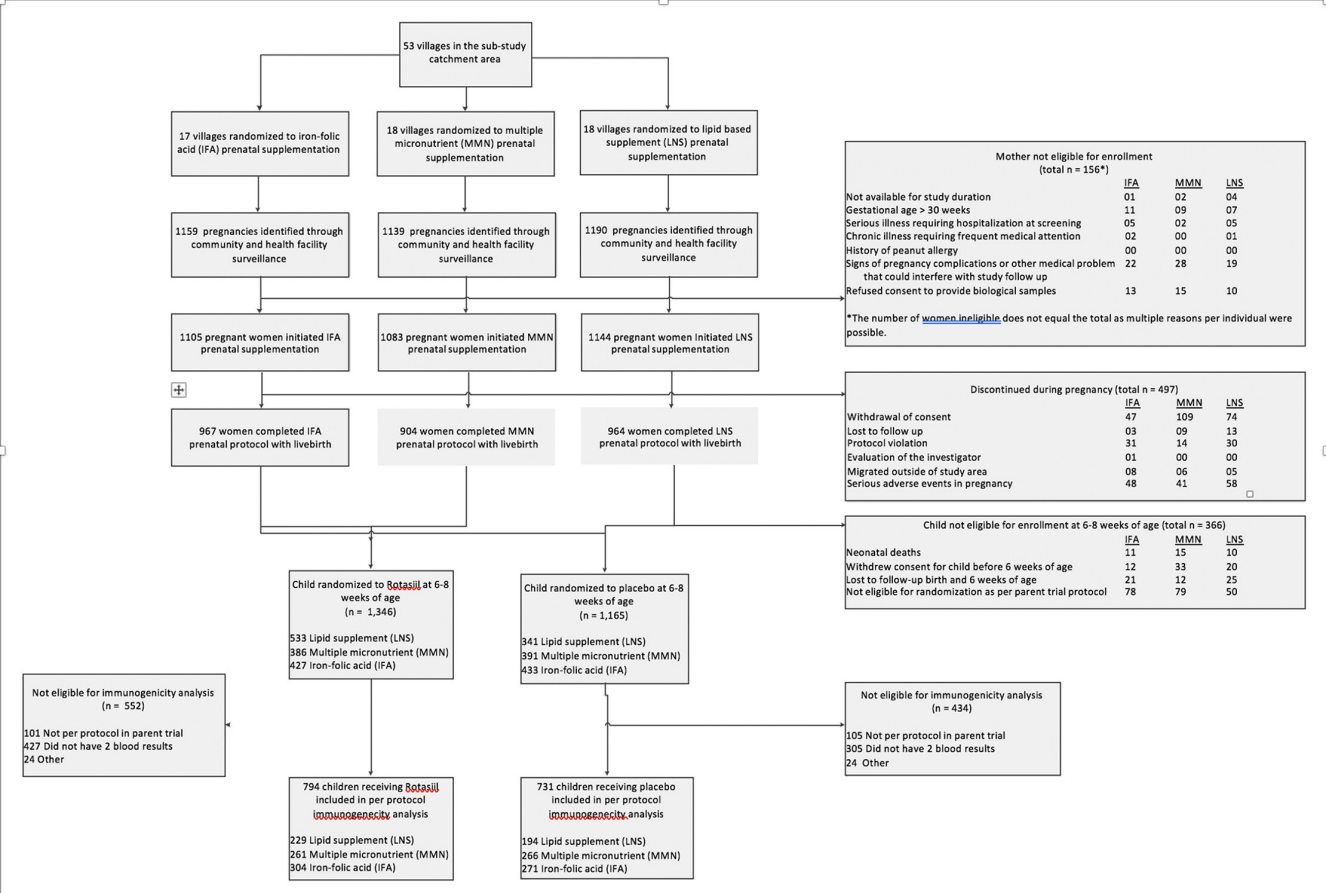
Flowchart of study participants. IFA, iron–folic acid; LNS, lipid-based nutrient supplement; MMN, multiple micronutrients.

A total of 53 villages were randomized to one of 3 prenatal nutritional supplements (Fig 1). Of the 3,341 pregnant women initiating prenatal nutritional supplementation in these villages, 2,844 (85%) completed the prenatal supplementation protocol with a live birth. Moreover, 87% of live-born children (*n* = 2,478) were confirmed eligible for randomization in the parent trial at 6 to 8 weeks of age, and the immunogenicity cohort comprised 1,525 infants with valid serology results (794 in the vaccine group and 731 in the placebo group). Demographic characteristics were similar between the vaccine and placebo groups and among the 3 prenatal supplement groups receiving vaccine (Table 1).

**Table 1:** Baseline characteristics of immunogenicity cohort.

	Rotasiil				Placebo
	All	IFA	MMN	LNS	
*N*	794	304	261	229	731
Age in months, mean (SD)					
Dose 1	6.30 (0.51)	6.36 (0.54)	6.27 (0.50)	6.24 (0.49)	6.27 (0.48)
Dose 2	10.36 (0.63)	10.43 (0.65)	10.33 (0.61)	10.29 (0.60)	10.32 (0.55)
Dose 3	14.40 (0.66)	14.47 (0.66)	14.38 (0.65)	14.32 (0.67)	14.38 (0.64)
Male, *n* (%)	393 (49.5)	145 (47.7)	140 (53.6)	108 (47.2)	377 (51.6)
Birth weight (kg), mean (SD)	3.11 (0.70)	3.02 (0.59)	3.17 (0.75)	3.17 (0.75)	3.15 (1.12)
Birth weight <2.5 kg, *n* (%)	69 (9.0)	26 (8.8)	16 (6.3)	27 (12.4)	65 (9.2)
Child weight (kg), mean (SD)					
Dose 1	4.55 (0.72)	4.57 (0.72)	4.55 (0.68)	4.55 (0.76)	4.61 (0.84)
Dose 2	5.34 (0.85)	5.33 (0.93)	5.29 (0.78)	5.41 (0.83)	5.34 (0.89)
Dose 3	5.92 (0.92)	5.86 (0.85)	5.93 (1.03)	5.99 (0.86)	5.89 (0.85)
Child height (cm), mean (SD)					
Dose 1	54.22 (2.58)	54.33 (2.42)	54.26 (2.53)	54.03 (2.83)	54.29 (2.62)
Dose 2	57.10 (3.47)	57.16 (3.34)	57.12 (3.56)	57.01 (3.53)	57.17 (3.02)
Dose 3	59.50 (2.82)	59.49 (3.06)	59.35 (2.79)	59.67 (2.53)	59.52 (2.65)
Any OPV coadministered on same day, *n* (%)					
Dose 1	253 (31.9)	102 (33.6)	67 (25.7)	84 (36.7)	233 (31.9)
Dose 2	214 (27.0)	85 (28.0)	61 (23.4)	68 (29.7)	215 (29.4)
Dose 3	176 (22.2)	64 (21.1)	58 (22.2)	54 (23.6)	152 (20.8)
Breastfed <30 minutes before dose, *n* (%)					
Dose 1	778 (99.5)	298 (99.0)	255 (100.0)	225 (99.6)	721 (100.0)
Dose 2	781 (99.6)	296 (99.3)	257 (100.0)	228 (99.6)	718 (99.9)
Dose 3	777 (99.6)	297 (99.7)	258 (99.6)	222 (99.6)	721 (99.9)
Breastfed <30 minutes after dose, *n* (%)					
Dose 1	779 (99.6)	299 (99.3)	255 (100.0)	225 (99.6)	720 (99.9)
Dose 2	783 (99.9)	297 (99.7)	257 (100.0)	229 (100.0)	718 (99.9)
Dose 3	776 (99.6)	296 (99.7)	258 (99.6)	222 (99.6)	721 (99.9)
Birth in health clinic, *n* (%)	408 (52.2)	164 (54.5)	142 (55.0)	102 (45.7)	365 (50.8)

IFA, iron–folic acid; LNS, lipid-based nutrient supplement; MMN, multiple micronutrient; OPV, oral polio vaccine.

### Immune response to Rotasiil with prenatal nutritional supplementation

Seroresponse to the vaccine did not differ by supplementation group: ≥3-fold rise in anti-RV IgA titers was detected in 41.1% (94/229) infants whose mothers received LNS compared with 39.1% (102/261) infants whose mothers received MMN and 38.8% (118/304) infants whose mothers received IFA (Table 2). The intraclass correlation coefficient was 0.005. There was no difference in GMC post-Dose 3 by group.

**Table 2:** Serum IgA seroconversion and mean concentration at Dose 1 and 28 days post-Dose 3 among vaccinated infants by prenatal supplementation group.

	IFA	MMN	LNS	*p*-value
≥3-fold response	*n* (%)	*n* (%)	*n* (%)	
	118 (38.8)	102 (39.1)	94 (41.1)	0.91
**GMC**	Mean (95% CI)	Mean (95% CI)	Mean (95% CI)	
*N*	304	261	229	
Pre-Dose 1	6.55 (5.63, 7.62)	6.93 (5.48, 8.75)	7.20 (5.69, 9.10)	0.66
28 days post-Dose 3	27.74 (23.16, 33.22)	29.23 (23.93, 35.69)	30.68 (23.38, 40.27)	0.81

CI, confidence interval; GMC, geometric mean concentration; IFA, iron–folic acid; IgA, immunoglobulin A; LNS, lipid-based nutrient supplement; MMN, multiple micronutrient.

### Anti-rotavirus IgA responses

Rotasiil was immunogenic with 39.6% (*n* = 314/794) of infants who received vaccine exhibiting a ≥3-fold rise in anti-rotavirus IgA compared to 29.0% (*n* = 212/731) of infants who received placebo (relative risk [RR]: 1.36; 95% CI: 1.18, 1.57; Table 3). At the time of receipt of Dose 1, 86.2% (*n* = 684) of the vaccine group and 86.2% (*n* = 630) of the placebo group were seronegative (IgA concentrations <20 AU/mL). The risk of a ≥3-fold rise was significantly greater among infants that were seronegative prior to Dose 1 (*p* for interaction = 0.006 RR: 1.46; 95% CI: 1.26, 1.70) and among infants that had the lowest anti-rotavirus IgG concentrations prior to Dose 1 (*p* for interaction = 0.006, Quartile 1 RR: 1.98; 95% CI: 1.53, 2.57). There was also evidence for a greater immune response among infants receiving Dose 1 outside of the peak rotavirus transmission season that generally occurs from October to February in Niger (*p* for interaction = 0.004; RR: 1.86; 95% CI: 1.42, 2.45).

At 28 days post-Dose 3, GMC for anti-rotavirus IgA among vaccinated infants was 29.0 AU/mL (95% CI: 25.4, 33.2) compared with 19.7 AU/mL (95% CI: 17.0, 22.8) among placebo recipients (GMC ratio: 1.47, 95% CI: 1.24–1.75). Differences in anti-rotavirus GMC post-Dose 3 were greatest among infants that were seronegative prior to Dose 1 (*p* for interaction = 0.005, GMC ratio: 1.59, 95% CI: 1.38 to 1.84), infants that had the lowest IgG anti-rotavirus concentrations prior to Dose 1 (*p* for interaction = 0.012, Quartile 1 GMC ratio: 2.48, 95% CI: 1.81 to 3.40), and among infants receiving Dose 1 outside of the peak rotavirus transmission season (*p* for interaction = 0.04; RR: 1.76; 95% CI: 1.12, 1.40).

**Table 3:** Serum IgA seroconversion and mean concentration at Dose 1 and 28 days post-Dose 3.

	Rotasiil	Placebo	
≥3-fold response	*N* (%)	*N* (%)	RR (95% CI)
All	314 (39.6)	212 (29.0)	1.36 (1.18, 1.57)
By child IgG quartile at pre-Dose 1			
Quartile 1	114 (56.7)	52 (28.9)	1.98 (1.53, 2.57)
Quartile 2	80 (39.2)	63 (35.0)	1.12 (0.86, 1.46)
Quartile 3	73 (37.6)	53 (28.5)	1.33 (1.00, 1.79)
Quartile 4	46 (23.7)	43 (23.5)	1.01 (0.70, 1.45)
By baseline child IgA			
≥20 AU/ml	24 (21.8)	30 (29.7)	0.71 (0.45, 1.11)
<20 AU/ml	290 (42.4)	182 (28.9)	1.46 (1.26, 1.70)
By season of Dose 1			
October to February (peak season)	194 (46.9)	152 (40.2)	1.17 (0.99, 1.37)
March to September (nonpeak)	120 (31.6)	60 (17.0)	1.86 (1.42, 2.45)
**GMC**	Mean (95% CI)	Mean (95% CI)	GMC ratio (95% CI)
All			
*N*	794	731	
Pre-Dose 1	6.84 (5.98, 7.83)	6.82 (5.89, 7.90)	1.00 (0.84, 1.19)
28 days post-Dose 3	29.00 (25.35, 33.19)	19.73 (17.04, 22.84)	1.47 (1.24, 1.75)
By baseline child IgA level			
Child baseline ≥20 AU/ml			
*N*	110	101	
Pre-Dose 1	166.0 (121.0, 227.9)	158.3 (112.0, 223.9)	1.05 (0.70, 1.58)
28 days post-Dose 3	161.1 (117.4, 221.1)	186.7 (132.0, 264.0)	0.86 (0.57, 1.30)
Child baseline <20 AU/ml			
*N*	684	630	
Pre-Dose 1	4.06 (3.68, 4.49)	4.10 (3.67, 4.59)	0.99 (0.86, 1.15)
28 days post-Dose 3	21.82 (19.75, 24.11)	13.71 (12.25, 15.34)	1.59 (1.38, 1.84)
By baseline child IgG quartile			
Quartile 1 (<172.06)			
*N*	201	180	
Pre-Dose 1	5.10 (4.04, 6.45)	5.33 (4.12, 6.88)	0.96 (0.70, 1.31)
28 days post-Dose 3	41.76 (33.05, 52.76)	16.86 (13.04, 21.79)	2.48 (1.81, 3.40)
Quartile 2 (172.06 to <302.02)			
*N*	204	180	
Pre-Dose 1	6.80 (5.47, 8.44)	6.24 (4.80, 8.11)	1.09 (0.78, 1.53)
28 days post-Dose 3	29.04 (23.38, 36.07)	22.94 (17.65, 29.82)	1.27 (0.90, 1.78)
Quartile 3 (302.02 to <532.23)			
*N*	194	186	
Pre-Dose 1	6.70 (5.18, 8.68)	7.67 (5.71, 10.31)	0.87 (0.61, 1.26)
28 days post-Dose 3	28.07 (21.69, 36.35)	22.22 (16.54, 29.85)	1.26 (0.88, 1.82)
Quartile 4 (≥532.23)			
*N*	194	183	
Pre-Dose 1	9.55 (7.07, 12.88)	8.53 (6.32, 11.52)	1.12 (0.79, 1.59)
28 days post-Dose 3	20.08 (14.88, 27.10)	17.39 (12.89, 23.47)	1.15 (0.81, 1.64)
By season of Dose 1			
October to February (peak season)			
*N*	414	378	
Pre-Dose 1	6.87 (5.82, 8.12)	7.02 (5.81, 8.48)	0.98 (0.76, 1.26)
28 days post-Dose 3	40.09 (33.92, 47.37)	33.03 (27.34, 39.91)	1.21 (0.94, 1.56)
March to September (nonpeak)			
*N*	380	353	
Pre-Dose 1	6.87 (5.74, 8.22)	6.80 (5.63, 8.22)	1.01 (0.81, 1.27)
28 days post-Dose 3	20.55 (17.17, 24.58)	11.68 (9.67, 14.12)	1.76 (1.12, 1.40)

CI, confidence interval; GMC, geometric mean concentration; IgA, immunoglobulin A; IgG, immunoglobulin G; RR, relative risk.

## Discussion

We assessed the immunogenicity of 3 doses of an oral rotavirus vaccine and the effect of prenatal nutritional supplementation to improve immune response among vaccinated children. Immune response varied depending on previous exposure to rotavirus, and high clinical protection has been shown in this setting [16]. The type of prenatal supplementation had no effect on immune response in this study.

This study was conducted in a high rotavirus transmission setting. Moreover, 40% of vaccinated infants and 29% of infants receiving placebo seroconverted, frequencies similar to that previously reported in sub-Saharan Africa[20] and India [10,21] but lower than in Western Europe [22]. The observation that 14% of infants that received placebo were seropositive (IgA ≥20 AU/ml) prior to Dose 1 at 6 to 8 weeks of age and 38% by 28 days post-Dose 3 at approximately 18 weeks of age highlight the high rotavirus circulation and very early natural infection in this setting. While it is difficult to assess immunogenicity in a high transmission setting, it is these settings where vaccines are needed most. In order to bring the benefits of vaccination to the most vulnerable populations, other strategies such as alternative delivery schedules (e.g., early dosing and/or boosters) and parenteral formulations should be considered.

Lower efficacy to oral vaccines (including rotavirus, polio, and cholera) in low-income countries than in high-income countries [13] represents a persistent challenge to reduce morbidity and mortality among the most vulnerable populations. To date, the reasons for lower vaccine efficacy in low-income countries remain unclear but could include maternally derived antibodies acquired either transplacentally or via breastfeeding, the coadministration of OPV, micronutrient deficiencies, or enteric coinfections and other concurrent infection and enteropathy [13]. As development and maintenance of the immune cells that support vaccine response depend on an adequate supply of micronutrients, the role of micronutrient status in poor vaccine response is increasingly recognized and micronutrient supplementation has been considered as a potential adjunctive intervention to improve oral vaccine performance [23–29]. Poor maternal micronutrient status would be expected to result in a nutrient-deficient fetal environment that impairs development of a functional immune system in utero and in micronutrient deficiencies at birth [30–32]. Studies among Gambian and Bangladeshi infants suggested that nutritional status during fetal life and early infancy may be critical for immune development [33,34]. This study offers further insight into the potential role of early life nutritional status underlying the oral vaccine efficacy gap, demonstrating here no improvement in immune response with prenatal nutrient supplementation. Supporting early life nutritional status, however, could have positive effects in specific subgroups that this study was not powered to examine [35], but further study is warranted. Prenatal supplementation may also provide other benefits to both maternal health, birth outcomes, and child growth and development [36].

We found that seroconversion was consistently more frequent among children with less direct or indirect exposure to rotavirus prior to Dose 1, with greater rates of seroconversion among children with lower concentrations of IgA and maternally derived IgG and those enrolled outside of the peak transmission season in Niger. Maternal IgG is transported across the placenta during pregnancy (primarily in the first or second month of life) and provides term infants protection against rotavirus infection [37]. Our findings, however, suggest that maternal IgG may interfere with the immune response to the vaccine when the first dose was administered at 6 to 8 weeks of age when the levels of IgG may be high. Maternal antibodies may impair the infectivity of live-attenuated vaccine viruses in the gut and thus inhibit ability of the vaccine to induce a robust immune response among infants. Studies in Pakistan and Vietnam similarly found that rotavirus IgA seroconversion was reduced among participants with higher levels of pre-vaccination maternally derived IgG [38,39]. In South Africa, authors found that infants who failed to seroconvert in response to an oral rotavirus vaccine had significantly higher IgG titers pre-Dose 1 than those who seroconverted, although the second dose partially overcame the interference as levels of IgG had waned at a median age of 16 weeks [40]. Since maternal IgG decrease with a half-life of 3 to 4 weeks, delayed vaccination to avoid potential interference of maternal antibodies might improve oral vaccine immunogenicity [41]. The greater immune response observed among children with low IgA concentrations prior to Dose 1 in this study has been reported elsewhere [16] and suggests that vaccine administration before natural infection and rise of IgA may be beneficial. Early (birth or neonatal) immunization has been considered as a possible strategy in settings where the burden of rotavirus gastroenteritis is high in the first 6 months of life [42]. Any change to current immunization schedules (earlier to address early acquisition of infection or later to address potential interference of maternal antibody) may need to be country specific due to differences in age of peak incidence but should aim to maximize coverage to achieve the full benefit of vaccination.

Our study had some limitations. Serum IgA immune response to rotavirus vaccines is considered the best surrogate marker of protection available [43], and, along with serum neutralizing antibodies, is evaluated with all rotavirus vaccines. IgA has been shown to correlate with vaccine efficacy at the population level, and robust IgA immune responses have been observed in various Western populations ranging from 85% to 95% [6,7]. We note, however, that there is no immune correlate of protection for rotavirus vaccines. In our setting, vaccine efficacy with 3 doses of Rotasiil was high (66.7%, 95% CI: 49.9, 77.9 [16]), but immunogenicity as measured by IgA seroconversion was modest. We further note the absolute concentrations of serum anti-rotavirus IgA among infants with previous natural infection (baseline levels of serum anti-rotavirus IgA ≥20 AU/ml: 166.0 AU/ml in the vaccine group and 158.3 AU/ml in the placebo group) were higher than among unexposed infants after vaccination (post-Dose 3 levels of serum anti-rotavirus concentrations IgA = 21.82 AU/ml). This finding seems to suggest that the absolute increase in serum anti-rotavirus IgA may be greater in response to natural infection than to vaccination (and that the importance of relative versus absolute levels of immune response may depend on baseline exposure). Longitudinal investigations among children in Mexico City have suggested that protection due to natural infection could be comparable to that achieved through complete vaccination [44,45], but the implications of using serum anti-rotavirus IgA for the assessment of immunogenicity and efficacy in low-income countries are unclear and warrant further consideration. Serum anti-rotavirus IgA may be an important factor in the host defense mechanism, but probably only one of several effectors of protection. New vaccine development would benefit from a viable immune correlate of protection, as clinical trials powered for clinical efficacy end points are resource intensive. Therefore, for both financial and ethical reasons, a validated laboratory marker of clinical protection that provided a robust basis for extrapolation of clinical efficacy data would be useful, eliminating the need for using clinical end points in future trials and spurring licensing of new vaccines and identification of new strategies to improve performance. Further efforts to identify alternate correlates of protection and analytical methods are warranted, and future studies could consider using a combination of serological and stool shedding end points to assess vaccine take.

## Conclusions

In a setting of high burden of natural infection, this study showed a modest increase in immune response with 3 doses of Rotasiil but not affected by prenatal nutrient supplementation. While the significance of the reduced seroresponse in this setting is not well understood, high clinical protection has been reported, and the administration of Rotasiil is expected to result in a large clinical benefit due to robust efficacy. Vaccine recommendations in settings with poor rotavirus IgA response may be based on efficacy data wherever possible. To improve vaccine performance in low-income settings, a more complete understanding of the factors influencing vaccine efficacy and a reliable correlate of protection are needed to improve vaccination strategies and accelerate vaccine development. Potential targets for population-level intervention to improve immune response are needed. Future studies could investigate potential factors such as enteropathy, coinfection, and gut microbiota, recognizing that multiple factors are likely involved in the reduced efficacy of oral vaccines. Without improvement in oral vaccine performance, alternative approaches, such as parenteral immunization or alternative dosing schedules, could be considered.

## Supporting information

S1 CONSORT Checklist CONSORT Checklist. CONSORT, Consolidated Standards of Reporting Trials.
(PDF)

S1 Table Supplemental results including Table A. (DOCX)

S1 Protocol Trial protocol. (PDF)

S1 Statistical Analysis Statistical analysis plan. (PDF)

## Abbreviations

CI: confidence interval
CONSORT: Consolidated Standards of Reporting Trials
ELISA: enzyme-linked immunosorbent assay
EPI: Expanded Program on Immunization
GMC: geometric mean concentration
IFA: iron–folic acid
IgA: immunoglobulin A
IgG: immunoglobulin G
LNS: lipid-based nutrient supplement
MMN: multiple micronutrients
MMS: multiple micronutrient supplement
OPV: oral polio vaccine
RDA: recommended daily allowance
RR: relative risk
SRVGE: severe rotavirus gastroenteritis
