# Correction: Immunogenicity of an oral rotavirus vaccine administered with prenatal nutritional support in Niger: A cluster randomized clinical trial

**DOI:** 10.1371/journal.pmed.1003776

**Published:** 2021-10-15

**Authors:** Sheila Isanaka, Souna Garba, Brian Plikaytis, Monica Malone McNeal, Ousmane Guindo, Céline Langendorf, Eric Adehossi, Iza Ciglenecki, Rebecca F. Grais

Fig 1 contains a typo. Please see corrected Fig 1 here.

**Fig 1:**
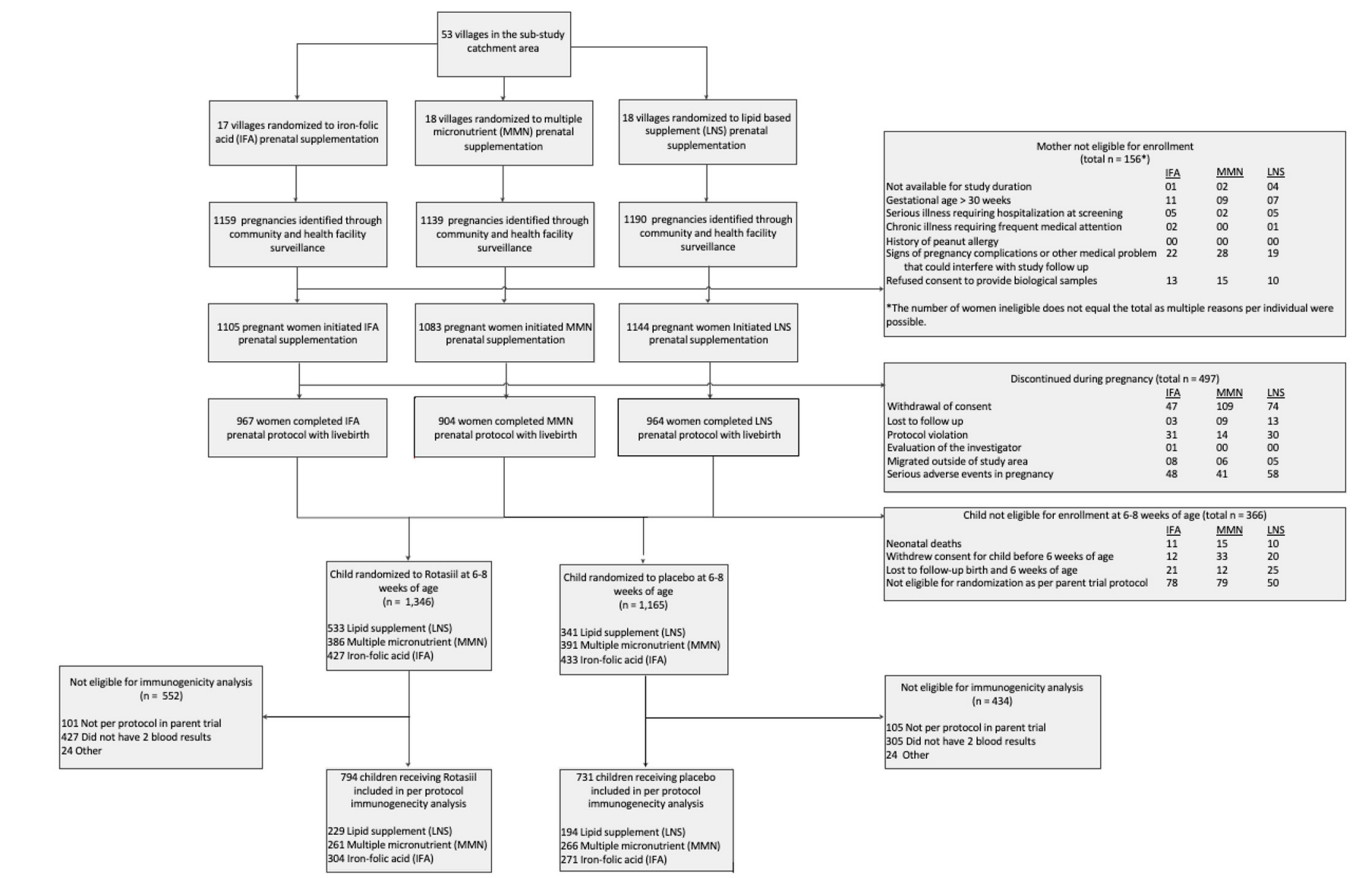
Flowchart of study participants. IFA, iron–folic acid; LNS, lipid-based nutrient supplement; MMN, multiple micronutrients. https://doi.org/10.1371/journal.pmed.1003720.g001.
