# Supplementary material for: Immunogenicity of an oral rotavirus vaccine administered with prenatal nutritional support in Niger: A cluster randomized clinical trial
Source: PLoS Med. 2021 Aug 10;18(8):e1003720. doi: 10.1371/journal.pmed.1003720 (PMC8354620; doi:10.1371/journal.pmed.1003720)
Supplement: S1 Table — (DOCX) [file pmed.1003720.s002.docx]

S1. Nutritional composition of study supplements

|  | **US RDA/AI for**  **pregnant**  **women** | **IFA** | **MMN** | **LNS (40g)** |
| --- | --- | --- | --- | --- |
| Energy (kcal) | 2500 | .. | .. | 237 |
| Proteins (g) | 50 | .. | .. | 5.2 |
| Milk proteins (g) | .. | .. | .. | 1.7 |
| Lipids (g) | .. | .. | .. | 20 |
| LA (*Linoleic Acid*) (g) | .. | .. | .. | 6.9 |
| ALA (*α-Linolenic Acid*) (g) | .. | .. | .. | 1.16 |
| Calcium (mg) | 1000 | .. | 559 | 559 |
| Phosphorus (*free*) (mg) | 700 | .. | 400 | 400 |
| Potassium (mg) | 4700 | .. | 400 | 400 |
| Magnesium (mg) | 350 | .. | 130 | 130 |
| Zinc (mg) | 11 | .. | 30 | 30 |
| Copper (mg) | 1 | .. | 4 | 4 |
| Iron (mg) | 27 | 60 | 30 | 30 |
| Manganese (mg) | 2 | .. | 2.6 | 2.6 |
| Iodine (µg) | 220 | .. | 250 | 250 |
| Selenium (µg) | 60 | .. | 130 | 130 |
| Vitamin A (µg) | 800 | .. | 800 | 800 |
| Vitamin B_1_ (mg) | 1.4 | .. | 2.8 | 2.8 |
| Vitamin B_2_ (mg) | 1.4 | .. | 2.8 | 2.8 |
| Niacin B_3_ (mg) | 18 | .. | 36 | 36 |
| Pantothenic acid - B_5_(mg) | 6 | .. | 7 | 7 |
| Vitamin B_6_ (mg) | 1.9 | .. | 3.8 | 3.8 |
| Vitamin B_12_ (µg) | 2.6 | .. | 5.2 | 5.2 |
| Folic acid (µg) | 600 | 400 | 400 | 400 |
| Vitamin C (mg) | 85 | .. | 100 | 100 |
| Vitamin D_3_ (µg) | 15 | .. | 15 | 15 |
| Vitamin E (mg) | 15 | .. | 20 | 20 |
| Vitamin K_1_ (µg) | 90 | .. | 45 | 45 |
